# Engineered HA hydrogel for stem cell transplantation in the brain: Biocompatibility data using a design of experiment approach

**DOI:** 10.1016/j.dib.2016.11.069

**Published:** 2016-11-24

**Authors:** Lina R. Nih, Pouria Moshayedi, Irene L. Llorente, Andrew R. Berg, Jessica Cinkornpumin, William E. Lowry, Tatiana Segura, S. Thomas Carmichael

**Affiliations:** a Department of Chemical and Biomolecular Engineering, University of California, 420 Westwood Plaza, Los Angeles, CA 90095, USA; b Department of Neurology, David Geffen School of Medicine, University of California, 635 Charles Young Drive, Los Angeles, CA 90095, USA; c Department of Molecular Cell and Developmental Biology, University of California, 710 Westwood Plaza, Los Angeles, CA 90095, USA

**Keywords:** Hydrogel, Hyaluronic acid, Hyaluronan, Brain, Brain repair, Stroke, Ischemia, Design of experiment, DOE, Biocompatibility, Toxicity, Stem cell transplantation, Neural stem cell, NPC, Astrocytic scar, Heparin, RGD, YIGSR, IKVAV, BDNF, BMP-4, Brain derived-neurotrophic factor, Bone-morphogenic protein-4

## Abstract

This article presents data related to the research article “Systematic optimization of an engineered hydrogel allows for selective control of human neural stem cell survival and differentiation after transplantation in the stroke brain” (P. Moshayedi, L.R. Nih, I.L. Llorente, A.R. Berg, J. Cinkornpumin, W.E. Lowry et al., 2016) [1] and focuses on the biocompatibility aspects of the hydrogel, including its stiffness and the inflammatory response of the transplanted organ. We have developed an injectable hyaluronic acid (HA)-based hydrogel for stem cell culture and transplantation, to promote brain tissue repair after stroke. This 3D biomaterial was engineered to bind bioactive signals such as adhesive motifs, as well as releasing growth factors while supporting cell growth and tissue infiltration. We used a Design of Experiment approach to create a complex matrix environment *in vitro* by keeping the hydrogel platform and cell type constant across conditions while systematically varying peptide motifs and growth factors. The optimized HA hydrogel promoted survival of encapsulated human induced pluripotent stem cell derived-neural progenitor cells (iPS-NPCs) after transplantation into the stroke cavity and differentially tuned transplanted cell fate through the promotion of glial, neuronal or immature/progenitor states. The highlights of this article include: (1) Data of cell and bioactive signals addition on the hydrogel mechanical properties and growth factor diffusion, (2) the use of a design of Experiment (DOE) approach (M.W. 2 Weible and T. Chan-Ling, 2007) [2] to select multi-factorial experimental conditions, and (3) Inflammatory response and cell survival after transplantation.

**Specifications Table**

**Table:** 

Subject area	Stem cell, Biology, Engineering, Material Sciences
More specific subject area	Biomaterial, Stem cell transplantation, brain repair
Type of data	Graph, figure, table
How data was acquired	Rheology, Elisa, Microscopy
Data format	Analyzed
Experimental factors	Cell encapsulation in hydrogel before transplantation
Experimental features	Human neural progenitor cell, 3D culture and brain transplantation
Data source location	Los Angeles, California, USA
Data accessibility	Data is provided in the article

**Value of the data**

• This work brings a deeper understanding on the influence of cell encapsulation and bioactive signal addition on the mechanical properties of the transplanted hydrogel and its growth factor diffusion.

• The data in this article show how a design of experiment (DOE) approach can be used to selectively choose multiple conditions in a multi-factorial experimental system.

• The *in vivo* data in this article highlight the influence of the different combinations of hydrogels on the brain inflammatory response and survival of encapsulated neural progenitor cells.

## 1 Data

**Fig. 1:**
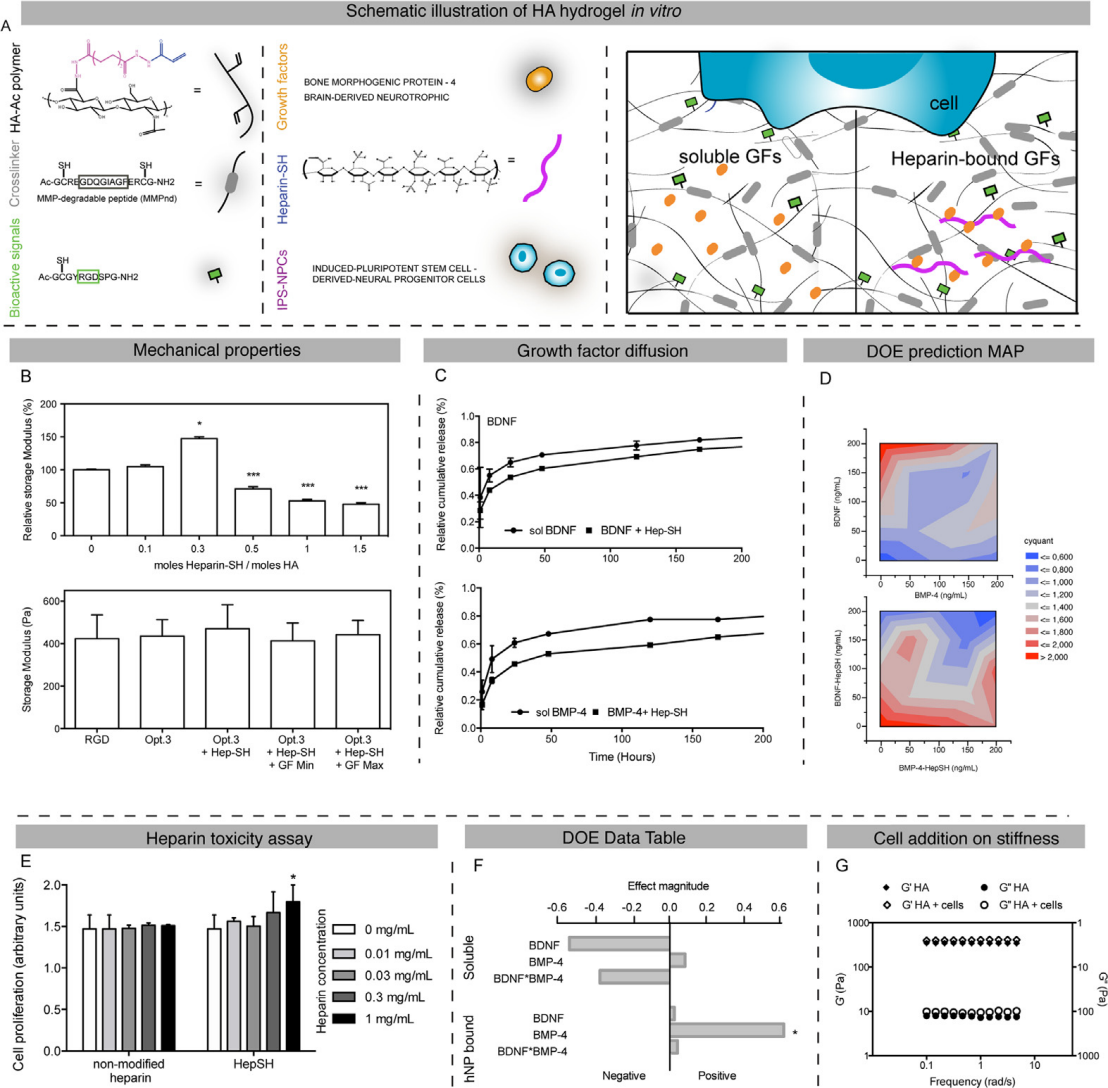
Mechanical and composition gel optimization *in vitro*. (A) Schematic illustration of the injectable hyaluronic acid (HA) composed of acrylated hyaluronic acid, MMP degradable or non-degradable motifs, adhesion peptides and heparin bound growth factors. (B) Rheology data evaluating the influence of heparin and adhesion motifs addition on HA gel stiffness. (C) Growth factor diffusion over time, after encapsulation in an HA-RGD gel. (D) DOE heat map showing the zones of cell survival on encapsulated iPS-NPCs in HA gel with the addition of soluble or heparin-bound BDNF and BMP-4 at day 7. (E) Heparin toxicity assay on 2D cell culture exposed to increasing concentrations of modified or non-modified heparin. (F) DOE data table showing the combination of heparin and growth factor leading to the most positive cell survival data in 3D at day 7. (G) Rheology data evaluating the influence of cell addition on gel stiffness. * indicates *P*<0.05.

**Fig. 2:**
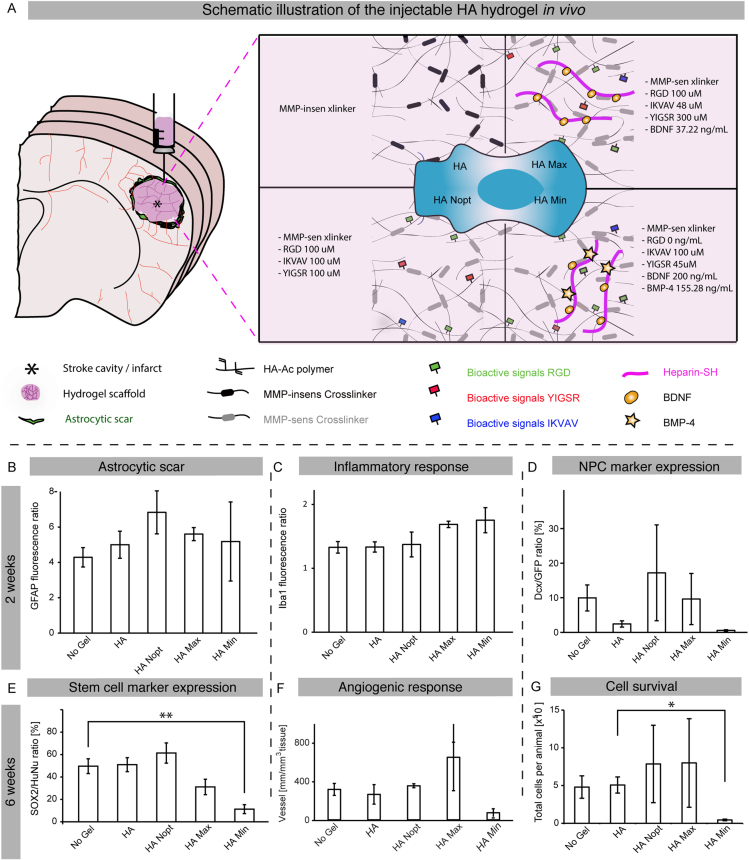
Biocompatibility data *in vivo*. (A) Schematic illustration of a brain coronal section after a cortical stroke and the transplanted hydrogel conditions. (B) Evaluation of the astrocytic scar density, (C) the microglial response and (D) the number of transplanted cells positive for the marker DCX, expressed in neuronal progenitors, 2 weeks after transplantation. (E) Evaluation of Sox2 expression, a stem cell marker, in transplanted cells, (F) quantification of vessel density in the stroke area and (G) Cell survival after gel transplantation at 6 weeks after stroke. * and ** indicate *P*<0.05 and *P*<0.01 respectively.

A hyaluronic acid hydrogel crosslinked *in situ* via thiol/acrylate Michael type addition, was used for human induced pluripotent neural precursor (iPS-NPC) 3D culture *in vitro,* and for a direct brain transplantation *in vivo, within the site of ischemic damage as previously described* [3]. Fig. 1 shows data related to the influence of heparin addition on mechanical properties, on cell toxicity and cell survival *in vitro*, while Fig. 2 presents dataset related to the *in vivo* transplantation of the cell and heparin-encapsulated hydrogel in stroked mice brain.

The gel was loaded with RGD and heparin both chemically modified to contain thiol groups and bind the HA backbone (Fig. 1A). In order to match the mechanical properties of brain cortex, the thiol/acrylate ratio as well as the HA weight % were modified to obtain a storage modulus of 350–400 Pa [4].

In this article, the influence of heparin addition on gel stiffness was evaluated on increasing concentrations of heparin, showing a significant increase of the storage modulus at a concentration of 0.3 mg/mL while higher concentrations of heparin were associated with a decreased storage modulus (Fig. 1B). Similarly, the influence of adhesion motifs on gel stiffness was evaluated on gels containing or not 0.3 mg/mL of heparin. The data show no significant difference between groups. Heparin addition on growth factor diffusion was then evaluated on HA hydrogels containing RGD and one of the neural growth factor brain derived-neurotrophic factor (BDNF) or bone-morphogenic protein-4 (BMP-4), showing a slower diffusion with the addition of heparin (Fig. 1C).

In order to evaluate the combined administration of the two growth factors on neural cell survival *in vitro*, Induced pluripotent stem cell-derived (iPS)-NPCs were encapsulated in HA-RGD-heparin gels and exposed to combinations of BDNF and BMP-4 concentrations rigorously selected using a statistical Design of Experiment (DOE) approach [5]. A heat map was obtained for both heparin and non-heparin conditions, showing that the greatest cell survival is obtained with high concentrations of BDNF and low concentrations of BMP-4, or low concentrations of both growth factors in the presence of heparin (Fig. 1D).

The influence of heparin addition alone, both modified and un-modified, on cell survival was then evaluated by exposing 2D cells to increasing concentrations of heparin. No significant difference was observed at a concentration of 0.3 mg/mL (Fig. 1E), while the combination of heparin and BMP-4 showed the greatest iPS-NPCs survival compared to any other combination (Fig. 1F). Finally, the influence of cell addition on gel stiffness was evaluated on HA-RGD gels and no significant difference was observed with and without cells (Fig. 1G).

The same statistical approach was used to optimize adhesion peptides derived from laminin (IKVAV and YIGSR) and fibronectin (RGD) to promote *in vitro* survival of iPS-NPCs and demonstrated that the optimal condition promoted neuronal differentiation [3]. A final stage of optimization consisted of combining both optimal formulations for growth factors and peptides [5] described as HA Max for the combination leading to the greatest survival, and HA Min for the minimal cell survival (Fig. 2A). The HA Min and Max conditions were selected for *in vivo* testing and compared to the following controls: (1) No gel, [2] an HA crosslinked with an MMP-insensitive crosslinker (HA), and (3) a non-optimized HA gel crosslinked with an MMP-sensitive crosslinker and equimolar concentrations of RGD, IKVAV and YIGSR (HA Nopt) (Fig. 2A). The five gel conditions were injected directly into the stroke cavity of mice brain. Brains were harvested and processed for sectioning and immunofluorescent staining 2 weeks later.

The staining for the astrocytic scar (GFAP) and microglia (Iba-1) 2 weeks after stroke showed no significant difference between conditions regarding the inflammatory response of the brain after gel injection (Fig. 2B, C). Similarly, no significant difference was observed with the number of IPS-NPCs positive for the marker Dcx at this time point (Fig. 2D).

The stem/progenitor cell expression pattern at 6 weeks after stroke was evaluated by the number of Sox2 positive human cells in the stroke area and showed a significantly reduced number in the HA Min condition compared with the HA group (Fig. 2E). A quantification of vessel density in the stroke area showed no significant difference between conditions (Fig. 2F).

Finally, the effect of the different gel combinations on cell survival after transplantation was assessed by evaluating the total number of human cell present per animal and a significantly reduced number of cells was observed in the HA Min condition compared with the HA group (Fig. 2G).

## 2 Experimental design, materials and methods

### 2.1 Cell culture

Induced pluripotent stem cells (iPS) were obtained from human fibroblasts [6] and directly differentiated in 2D culture from iPS through formation of neural rosettes [7]. On the day of transplantation, human iPS-NPCs were harvested by a 5 min incubation in presence of TrypLE (Life Technologies), centrifuged at 300 g for 5 min, re-suspended in animal origin-free culture medium and kept on ice before injection.

### 2.2 Hyaluronic acid modification

Hyaluronic acid (HA- 60,000 Da, Genzyme, Cambridge, MA) was modified as previously described [1]. Briefly, HA was functionalized with an acrylate group using a two-step synthesis as previously described [8]. HA (2.0 g, 5.28 mmol) was dissolved in water and reacted with adipic dihydrazide (ADH, 18.0 g, 105.5 mmol) in the presence of 1-ethyl-3-(dimethylaminopropyl) carbodiimide hydrochloride (EDC, 4.0 g, 20 mmol) for 8 h at a pH of 4.75. The hydrazide-modified HA (HA-ADH) was then purified in presence of decreasing amounts of NaCl (100, 75, 50 and 25 mmol) for 4 h each via dialysis (8000 MWCO). A purification in deionized water for 48 h and a lyophilization followed. A resuspension of HA-ADH in 4-(2-hydroxyethyl)-1-piperazine ethane-sulfonic acid (HEPES) buffer (10 mM HEPES, 150 mM NaCl, 10 mM EDTA, pH 7.4) and a reaction with N-acryloxysuccinimide (NHS-AC), 1.33 g, 4.4 mmol) was performed overnight, before purification as described earlier, and lyophilization. A final product, acryl hydrazide hyaluronic acid (HA-AC) was obtained.

The degree of acrylation (14.9%) of the final product was determined usong 1H NMR and by dividing the multiplet peak at δ=6.2 (cis and trans acrylate hydrogens) by the singlet peak at δ=1.6 (singlet peak of acetyl methyl protons in HA). The specific selection of HA as a hydrogel relies on its biocompatibility with human tissue, as it is constituted of naturally occurring brain extracellular matrix constituents. Furthermore, HA has been shown to enhance vessel growth in wound sites and to promote repair [5], [9]. The final purified product was lyophilized and stored at 4 °C until used.

### 2.3 Heparin modification

Heparin Carboxylic acid groups (Alfa Caesar, Ward Hill, MA) were functionalized with thiol groups by reacting heparin to EDC, NHS, and cysteamine while maintaining the pH at 4.75. The reduction of oxidized disulfide groups was performed by the addition of 1,4-Dithiothreitol to the final product overnight at pH 7.5 and then adjusted to pH 3.5. The obtained product was purified by dialysis and lyophilization. An Ellman׳s reaction at 412 nm was used to determine the amount of thiol group bound to heparin.

### 2.4 Gelation

HA-AC was mixed with HEPES buffer for 15 min at 37 °C for a complete dissolution. The appropriate concentration of Ac-GCGYGRGDSPG-NH2 adhesion peptide (RGD, Genscript, Piscataway, NJ), Ac-GCGYGYIGSR-NH2 (YIGSR, Genscript), Ac-IKVAVGYGCG-NH2 (IKVAV, Genscript) was dissolved in HEPES and added to the dissolved HA-AC and allowed to react for 20 min. A total concentration of (3000 iPS-NPCs/µL was used a final concentration for *in vitro* experiments, Heparin and growth factors were then added to the gel precursor solution, before the addition of a MMP-degradable peptide cross-linker (Ac- GCRDGPQGIWGQDRCG-NH2, Genscript), dissolved in 0.3 M HEPES. A total of 5 µL of this solution was pipetted onto, and sandwiched between two Sigmacote (Sigma-Aldrich) functionalized glass coverslips and placed in an incubator for 30 min at 37 °C to gel. For *in vivo* experiments, the precursor was loaded into the Hamilton syringe directly after mixing in the desired cross-linking peptide.

### 2.5 Rheometry

Gels were placed in a plate-to-plate rheometer (Physica MCR 301, Anton Paar, Ashland, VA) with a frequency range of 0.1–10 rad/s under a constant strain of 1% at 37 °C.

### 2.6 Growth factor diffusion

Gels with or without heparin were loaded with BDNF or BMP-4 (1 µg/mL) and incubated in PBS. The solution was collected every hour then every day and the concentration of growth factor was measured by ELISA (R&D systems, Minneapolis, MN).

### 2.7 Cell survival in vitro

IPS-NPC survival at 1 and 7 days after gel gelation was measured using the CyQUANT kit (Invitrogen). Gels were quickly degraded using in TrypLE (Invitrogen) at 37 °C and centrifuged at 250 g for 5 min. Cells were re-suspended in 200 µL of the CyQUANT solution and the fluorescence was read at 480 nm.

### 2.8 Design of experiments

A surface response methodology [10] was used to create a range of factor of interest using a central composite inscribed design (CCI). For each iteration, a response surface methodology experiment was developed in which each variable factor was given a defined range (µM) to be modulated within. Here, the Central composite on face, a specific RSM statistical design was chosen, as this system enabled us to test the end points of each factor. Data from the cell survival assay for each condition recommended was inputted back into the software. The data was analyzed via a least squares regression model to determine significance of the factors and plot the predicted response surface. Each condition was done in triplicate and data from gels at day 7 were normalized to gels from the same condition at day 1 to account for any differences in the numbers of cells encapsulated in the gel precursor solution.

### 2.9 Inducing stroke and transplantation

The procedures involving animals and human cells were performed in accordance with National Institutes of Health Animal Protection Guidelines and approved by the UCLA Chancellor׳s Animal Research Committee as well as the UCLA Office of Environment Health and Safety. A cortical model of photothrombotic stroke [11] was produced in 8-weeks male C57BL/6 mice (Jackson Laboratory, Bar Harbor, ME) or Nod-Scid-gamma (NSG) mice (Jackson Laboratories). Stroke was produced under 2% isoflurane in N2O:O2 (2:1) as previously described [1].

At day 7 after stroke, when the rapid cell death left an empty cavity in the infarcted tissue [12], cells at a concentration of 50,000 cells/µL, gel (2 µL) or both were injected into compartmentalized wound using a 25 µL Hamilton syringe (Hamilton Company, Reno, NV) and a 30 G Hamilton needle into a point 0.6 mm below the surface of the brain at 0.3 µL/mL rate. The cell survival data in C57BL/6 mice were performed on immunosuppressed animals with Tacrolimus (Cell Signaling, Danvers, MA). Four intraperitoneal injections of Tacrolimus (3 mg/kg; dissolved in DMSO) were delivered for total of 4 days starting 2 days before implanting minipumps. At the time of the stroke surgery, 3 mg/kg/day Tacrolimus (dissolved in 50% DMSO, 15% ethanol and 35% distilled water) was infused from a minipump (Model 1002; DURECT Corporation, Cupertino, CA). Minipumps were implanted subdermally between the scapulae and changed every two weeks. Upon minipump extraction a successful infusion of Tacrolimus was ensured by checking for any remaining Tacrolimus solution. Additional subcutaneous injections of tacrolimus were administered on the day of pump replacement since it takes ~12 h for miniosmotic pump to start infusion.

### 2.10 Tissue processing

The infarct size and remaining hydrogel volume was determined 4 days after stroke using 2% triphenyl tetrazolium chloride (TTC; gr/mL, made in PBS; Sigma-Aldrich, St Louis, MO) solution on brain sections of 1 mm thick as previously described [1]. For immunohistochemical data (Fig. 2), 16 µm thick paraformaldehyde-fixed brain sections were stained at 2 and 6 weeks after stroke using primary antibodies (Supplemental data Table S1) in blocking buffer overnight at +4 °C followed by fluorophore-conjugated secondary antibodies in PBS for 1 h at room temperature.

### 2.11 Tissue analysis

Stained slides were scanned using confocal microscopy (C2 Series, Nikon Instruments Inc., Melville, NY) and positively labeled signal measured by ImageJ (ImageJ v1.43, Bethesda, Maryland, USA) and IMARIS (Bitplane, South Windsor, CT) softwares.

### 2.12 Statistics

A Power analysis tool (Statistical Solutions LLC, Cottage Grove, WI) was used to determine the animal sample size with the expected variance based on preliminary data. Data for each group at each time point comprise 4–7 animals. Data is presented as mean±SEM. Multiple comparisons ANOVA 1 way with Bonferonni or Dunnet post-hoc tests were performed. When data did not fit into a normal distribution, tests appropriate for non-parametric variables, such as Mann–Whitney or Kruskal–Wallis test and Dunn post-hoc test were employed.

## Footnotes

Appendix A Supplementary data associated with this article can be found in the online version at 10.1016/j.dib.2016.11.069.
