# Supplementary material for: Engineered HA hydrogel for stem cell transplantation in the brain: Biocompatibility data using a design of experiment approach
Source: Data Brief. 2016 Nov 24;10:202–9. doi: 10.1016/j.dib.2016.11.069 (PMC5154973; doi:10.1016/j.dib.2016.11.069)
Supplement: Supplementary file 1 — Supplementary material [file mmc1.docx]

Conflict of interest : None
