# Supplementary material for: Engineered HA hydrogel for stem cell transplantation in the brain: Biocompatibility data using a design of experiment approach
Source: Data Brief. 2016 Nov 24;10:202–9. doi: 10.1016/j.dib.2016.11.069 (PMC5154973; doi:10.1016/j.dib.2016.11.069)
Supplement: Supplementary file 2 — Supplementary material [file mmc2.pdf]

Supplementary Data

#	Antigen	Source	Company	Cat#	Dilution
1	CD31	rat	BD Pharmingen	553370	1:200
2	DCX1	guinea pig	Milipore	AB2253	1:2000
3	GFAP	rat	Life Sciences	13-0300	1:500
4	GFP	goat	Gift	Gift	1:500
5	HuNu	mouse	Millipore	MAB1281	1:500
6	Iba1	rabbit	Wako	019-19741	1:500
12	SOX2	rabbit	Cell Signaling	3579	1:300

Table S1. Antibody specifications
